# An auditory perspective on phonological development in infancy

**DOI:** 10.3389/fpsyg.2023.1321311

**Published:** 2024-01-24

**Authors:** Monica Hegde, Thierry Nazzi, Laurianne Cabrera

**Affiliations:** Integrative Neuroscience and Cognition Center (INCC-UMR 8002), Université Paris Cité-CNRS, Paris, France

**Keywords:** infants, amplitude modulations, frequency modulations, vocoder, speech perceptual attunement, phonetic processing

## Abstract

**Introduction:**

The auditory system encodes the phonetic features of languages by processing spectro-temporal modulations in speech, which can be described at two time scales: relatively slow amplitude variations over time (AM, further distinguished into the slowest <8–16 Hz and faster components 16–500 Hz), and frequency modulations (FM, oscillating at higher rates about 600–10 kHz). While adults require only the slowest AM cues to identify and discriminate speech sounds, infants have been shown to also require faster AM cues (>8–16 Hz) for similar tasks.

**Methods:**

Using an observer-based psychophysical method, this study measured the ability of typical-hearing 6-month-olds, 10-month-olds, and adults to detect a change in the vowel or consonant features of consonant-vowel syllables when temporal modulations are selectively degraded. Two acoustically degraded conditions were designed, replacing FM cues with pure tones in 32 frequency bands, and then extracting AM cues in each frequency band with two different low-pass cut- off frequencies: (1) half the bandwidth (Fast AM condition), (2) <8 Hz (Slow AM condition).

**Results:**

In the Fast AM condition, results show that with reduced FM cues, 85% of 6-month-olds, 72.5% of 10-month-olds, and 100% of adults successfully categorize phonemes. Among participants who passed the Fast AM condition, 67% of 6-month-olds, 75% of 10-month-olds, and 95% of adults passed the Slow AM condition. Furthermore, across the three age groups, the proportion of participants able to detect phonetic category change did not differ between the vowel and consonant conditions. However, age-related differences were observed for vowel categorization: while the 6- and 10-month-old groups did not differ from one another, they both independently differed from adults. Moreover, for consonant categorization, 10-month-olds were more impacted by acoustic temporal degradation compared to 6-month-olds, and showed a greater decline in detection success rates between the Fast AM and Slow AM conditions.

**Discussion:**

The degradation of FM and faster AM cues (>8 Hz) appears to strongly affect consonant processing at 10 months of age. These findings suggest that between 6 and 10 months, infants show different developmental trajectories in the perceptual weight of speech temporal acoustic cues for vowel and consonant processing, possibly linked to phonological attunement.

## 1 Introduction

The auditory system encodes the phonetic features of a given language by processing fine spectro-temporal acoustic changes in the speech signal. Even with a relatively immature auditory system (Moore, 2002), infants have been shown to distinguish phonetic contrasts in a language-specific manner before the end of their first year of life (see Kuhl, 2004; Saffran et al., 2006). However, it remains unclear whether infants and adults rely on the exact same acoustic information when discriminating native phonetic contrasts. To this aim, the current study compares the reliance upon spectro-temporal acoustic cues of speech in a phonetic feature discrimination task between infants at two ages (6 and 10 months) and adults. This study aims to investigate whether infants at different developmental stages, as well as adults, use the same acoustic information to discriminate vowels and consonants in their native language.

To explore infants auditory processing of speech, the present study uses a psychoacoustic approach that has been described extensively over the last decades and modeled the stages of auditory processing in adult listeners (c.f., Moore and Linthicum, 2007). A key concept of this psychoacoustic approach is to consider that the human auditory system decomposes any complex acoustic signal (including speech) into its fine spectral and its fine temporal modulations. The decomposition of the spectral modulations is related to the sensitivity of inner hair cells within the basilar membrane of the cochlea to a specific audio frequency range. The selective spectral processing of audio frequency from the high frequencies at the base of cochlea to low frequencies at the apex can be modeled as a bank of narrowband filters with a passband equal to one equivalent-rectangular bandwidth (ERB, Glasberg and Moore, 1990; Moore, 2003). Then, the auditory system is thought to decompose the temporal components of each extracted narrowband signal at two main time scales: relatively slow amplitude variations over time (amplitude modulations or AM, often referred to as temporal envelope), and relatively fast oscillations over time (frequency modulation or FM, often referred to as temporal fine structure). These models helped to develop speech analysis-synthesis tools, called vocoders, to assess selectively the specific role of spectral and temporal components in speech perception. Using vocoders, the spectro-temporal complexity of an original speech can be selectively manipulated.

In adults, a wealth of studies using vocoders showed that FM cues convey essential information related to voice pitch, and play an important role in speech perception in quiet for lexical-tone languages (using pitch at the syllable level, e.g., Zeng et al., 2005; Kong and Zeng, 2006). Moreover, sentence recognition has been found to be more difficult when only FM cues are preserved in the signal (Gilbert and Lorenzi, 2006; Lorenzi et al., 2006; Sheft et al., 2008; Hopkins et al., 2010), but FM cues provide crucial information in noisy environments (e.g., Zeng et al., 2005; Hopkins et al., 2008; Hopkins and Moore, 2009; Ardoint and Lorenzi, 2010). Nevertheless, AM cues have been found to convey information related to syllabic and phonetic information that allow word and sentence identification in quiet listening conditions (Rosen, 1992; Shannon et al., 1995; Smith et al., 2002; Zeng et al., 2005; Lorenzi et al., 2006; Sheft et al., 2008). This was initially demonstrated by Shannon et al. (1995) using noise-excited vocoders to investigate the impact of spectro-temporal degradation on speech identification. In that study, the researchers took original input sentences and applied a filter-bank to decompose the signal into 1, 2, 3, or 4 frequency bands from which the original AM and FM cues were decomposed. While the FM was replaced by a noise carrier in each band, the AM cues were low-pass filtered at different cutoff frequencies (16, 50, 160, or 500 Hz). Sentence identification scores in quiet were almost perfect in the 4 band-AM condition but decreased with a reduced number of frequency bands. Moreover, sentence recognition scores were worse in the condition where AM cues were preserved only below 16 Hz. Other studies showed that faster AM cues transmit some information regarding voice pitch information (Kong and Zeng, 2006) as well as formant transitions (Rosen, 1992).

While it has been repeatedly observed that adults are able to correctly identify speech in quiet with only the slowest AM cues (<8–16 Hz), the identification of individual phonetic features becomes more nuanced in terms of what acoustic cues are used. Using confusion matrices of phonemes, Shannon et al. (1995) showed that the reduction of faster AM cues (>16 Hz) significantly affected consonant identification, but not vowel identification. Moreover, for consonants, the identification of place of articulation remained challenging even in the 4-band AM condition. More recently, Xu et al. (2005) conducted a systematic study to determine the importance of various spectral and temporal information in phoneme identification. English-speaking adults were asked to identify consonants and vowels that varied in voicing, place of articulation, manner of articulation, duration, first formant (F1) frequency and second formant (F2) frequency. Syllables were vocoded using different numbers of bands (ranging from 1 to 16) and different low-pass filters for AM extraction (ranging from 1 to 512 Hz). Their findings showed that the optimal low-pass cutoff frequency for consonant recognition was 16 Hz, whereas for vowel recognition it was 4 Hz. Regarding spectral information, consonant recognition performance reached a plateau at 8 bands, while for vowel recognition it was 12 bands. These findings from adult studies show that AM cues are the most important cue for overall speech recognition in quiet (i.e., at the sentence recognition level), but that identification of consonants and vowels require different contributions of fast and slow AM, and FM cues. In other words, this demonstrates that various spectro-temporal cues play distinct functional roles in phoneme identification. However, it is important to note that these conclusions concern listeners with a mature auditory system and a well developed linguistic system.

To tackle developmental issues, vocoders have also been used to investigate how young listeners and especially infants use acoustic cues when processing speech sounds. Although this field of research is still largely emerging, the first infants studies using vocoders suggest that AM and FM cues have a different role at early ages compared to adults. For vowels, only one study to date has assessed English-learning 6-month-olds ability to detect a phonetic change in degraded speech. This study tested discrimination between /a/ and /i/ in vocoder conditions reducing FM cues and the number of spectral bands for AM extraction. Infants were found to detect a vowel change when the original AM (160 Hz cut-off frequency) was presented within 32 bands, but not when it was presented within 16 bands (Warner-Czyz et al., 2014). It is however not clear yet whether infants require faster fluctuations of AM to process vowels.

For consonants, on the other hand, a handful of studies have investigated phonetic discrimination in young infants. Two studies used looking-time recording procedures to familiarize or habituate French-learning infants to one specific vowel-consonant-vowel sequence processed in one vocoder condition. The findings reveal that 6-month-olds were able to distinguish /aba/ from /apa/ when the slowest (<16 Hz) AM cues were preserved in only 32 bands, but that they required an increased time of listening to display this behavior compared to a condition where the original (<ERB/2) AM cues were preserved (Cabrera et al., 2013, 2015a). These studies demonstrate that 6-month-old infants can effectively use slow (<16 Hz) AM cues for consonant voicing or place discrimination, but that faster AM cues may play an important role in early phonetic discrimination. Results along this line were also found in younger infants in a more recent study by Cabrera and Werner (2017) using an observer-based psychophysical procedure, the method used in the present study. English-speaking adult and English-learning 3-month-old participants were presented with one of five consonant categories (voiceless, voiced, labial, coronal, velar). In a yes-no task, participants were presented with a series of background syllables that exemplified the category under examination (e.g., voiced syllables like /ba/, /da/, /ga/, randomly repeated). They were evaluated based on their ability to detect change trials, where a single randomly selected “target” syllable (e.g., voiceless syllables like /pa/, /ta/, or /ka/) was played, and to withhold responses during no-change trials, where a background syllable was presented. Both infants and adults were tested on their ability to discriminate consonants under quiet or noisy conditions in two vocoder conditions: (1) Fast AM, in which the original AM (filtered < 256 Hz) was preserved in 32 bands and FM was replaced by a pure tone, and (2) Slow AM, in which only the slowest AM (filtered < 8 Hz) was preserved in 32 bands and FM was replaced by a pure tone. Adults were able to discriminate consonants in both vocoder conditions in quiet environments. However, in noisy environments, the percentage of adults who correctly discriminated the consonant changes decreased from 70% to 20% between the Fast and the Slow AM conditions. These results confirmed that the slowest AM cues are not sufficient for adults consonant discrimination in noise. Infants did not discriminate consonants equally in both vocoder conditions in quiet environments. The percentage of infants who discriminated decreased from 81% to 50% between the Fast and Slow AM conditions. In noisy environments, a similar pattern emerged, with the percentage of infants discriminating decreasing from 96% to 48% between the Fast and Slow AM conditions. In summary, these first infant studies using vocoders suggest that 3- and 6-month-old infants may not rely on exactly the same spectro-temporal modulations as adults when processing phonemes. However, the age at which infants start to use, or weight, the acoustic cues found to be used by adults to process speech remains unknown. This developmental shift must occur between early infancy and adulthood, and possibly when infants start to process speech sounds in a language-specific manner, that is, during the second half of the first year of life.

The present study aims to investigate the development of the early auditory processing of speech to provide further insights into the acquisition of the phonological properties specific to one's native language. Interestingly, during the first year of life, infants show asynchronous perceptual attunement to the vowels and the consonants of their native language. Specifically, infants start becoming attuned to native language vowels around 4–6 months of age (Trehub, 1976; Kuhl et al., 1992; Polka and Werker, 1994), earlier than when they start becoming attuned to native language consonants around 8-10 months of age (Trehub, 1976; Werker and Tees, 1984; Best et al., 1988, 1995). Furthermore, at the lexical level, differences in processing of vowels and consonants are also found, showing a shift in infants reliance from vowels to consonants between 6 and 8-11 months of age when detecting word forms (Bouchon et al., 2015; Poltrock and Nazzi, 2015; Nazzi et al., 2016; Nishibayashi and Nazzi, 2016). The question arises as to whether changes in spectro-temporal cue processing occur during this same developmental time window, and could thus be linked to phonological acquisition during the first year of life.

While no study to date has explored this issue directly, one study investigated the development of spectro-temporal cue weighting in a cross-linguistic study comparing French- versus Mandarin-learning infants. Cabrera et al. (2015b) investigated whether native language exposure influences reliance upon AM and FM cues in a discrimination task measuring looking times for two syllables varying in lexical tone (that is a change in pitch at the syllable level, such contrasts being phonological in tonal languages such as Mandarin Chinese, but not in French). Results showed that at 6 months, French- and Mandarin-learning infants display the same pattern of response: they detected a change in lexical tones in an intact condition (without acoustic degradation), suggesting that French-learning infants were not yet attuned to this speech contrast, and both groups did not detect the change when fine spectral and FM cues were degraded, showing that these acoustic cues are required for lexical-tone detection at 6 months. However, at 10 months, an influence of language background was observed: Mandarin-learning 10-month-olds showed the same pattern of response as 6-month-olds, but French-learning 10-month-olds were not able to detect the lexical-tone change in the intact condition, showing perceptual reorganization for this speech contrast. Moreover, French-learning 10-month-olds were able to discriminate the lexical tones when fine spectral and FM cues were degraded. These results suggest that native language exposure plays a role in the development of acoustic cue weighting during the phonological reorganization period.

Accordingly, the current study focused on infants of 6 and 10 months of age exposed to French and compares their reliance upon FM and AM cues when detecting native vowel or consonant feature contrasts to assess whether with age infants rely more on slow or faster temporal cues when processing native phonemes. The present study will extend the findings of Cabrera and Werner (2017), using an observer-based psychophysical yes-no task to measure the proportions of listeners able to detect a phonetic change in two vocoder conditions. Particularly, we compared the number of adults, 10-month-old and 6-month-old infants correctly detecting vowel or consonant changes in quiet based on various types of phonetic features, and in two vocoder conditions reducing increasingly FM and AM cues.

Three groups of participants were tested in the exact same experimental conditions and setup: 6-month-olds, who have started to attune to the vowels but not the consonants of their native language; 10-month-olds, who have started to attune to both vowels and consonants of their native language; and adults. Eight phonetic conditions were designed to assess the ability of listeners to detect a change in: Vowel Place, Vowel Height, Consonant Place and Consonant Voicing, each tested in two vocoder conditions, a Fast AM condition (preserving the original AM cues by using a cutoff frequency of ERB/2 that preserves fast and slow AM, in 32 bands, with reduced FM cues), and a Slow AM condition (preserving only the slowest AM cues below 8 Hz, in 32 bands, with reduced FM cues). Listeners were exposed to only one phonetic feature contrast in its two vocoder conditions, starting with the Fast AM condition and then, if they succeeded, moving to the Slow AM condition. Therefore, this study specifically examines: (1) the contributions of FM, Fast AM, and Slow AM cues for phonetic categorization (2) the role of these cues at distinct developmental points, (3) how these cues influence the categorization of vowels and consonants, and (4) the impact of different phonetic features in the aforementioned categorization.

Based on prior behavioral studies, we expected a higher success rate among 6-month-olds in the Fast AM condition compared to the Slow AM condition, as these infants typically exhibit a stronger weighting of Fast AM cues. Nonetheless, as 6-month-olds have already started to attune to the vowels of their native language, we hypothesized that temporal degradation may have a more pronounced effect on consonant detection than on vowel detection. For 10-month-olds, we predicted similar performance for both the Fast AM and Slow AM conditions, as they have started to attune to both vowels and consonants of their native language. As such, we expected any difference between the effect of temporal degradation on vowels and on consonants to be less pronounced in 10-month-olds than in 6-month-olds. For adults, we predicted near-ceiling performance in all conditions.

## 2 Methods

### 2.1 Participants

Participants were recruited through the Babylab Participant Pool at the Integrative Neuroscience and Cognition Center. The data of 40 6-month-old infants (mean: 28.2 weeks, range: 25.9 weeks–31.8 weeks; 24 girls, 16 boys), 40 10-month-old infants (mean: 45.9 weeks, range: 42.4 weeks–49.6 weeks; 16 girls, 24 boys) and 20 adults (mean: 21 years; range: 18 to 29 years; 13 females, 7 males) were included in the analyses. All infants were born full term, had no history of otitis media within 3 weeks of testing with no more than 2 prior occurrences of otitis media, had no risk factors for hearing loss, were French monolinguals (French input > 90% of the time) and had no history of health or developmental concerns. All adult participants were native French monolingual speakers, reported typical hearing bilaterally and had no history of noise exposure. Informed consent forms were obtained from all infants legal guardians and adult participants as approved by the university ethics committee. Data from an additional three 6-month-olds and two 10-month-old were excluded because the infants were too tired or fussy to complete the task; and data from three 6-month-olds and four 10-month-olds were excluded because parents did not come back for the second testing session.

### 2.2 Stimuli

A total of 16 Consonant-Vowel (CV) syllables were chosen so that by different recombinations, they could be used to define two vowel categories contrasted on place, or two vowel categories contrasted on height or two consonant categories contrasted on place, or two consonant categories contrasted on voicing. Each of these categories was made up of eight syllables, in which both vowels and consonants were varied (see Table 1). The 16 CV syllables used in the study are as follows: pu, bu, tu, du, po, bo, to, do, py, by, ty, dy, pø, bø, tø, and dø. The CV syllables were recorded in a sound-attenuated room and digitized with 16-bit resolution at a 44.1-kHz sampling rate. A female French native speaker who was instructed to “speak clearly” produced several tokens of all the CVs and five tokens for each were selected for their clarity. All tokens were comparable in duration (range = 263:411 ms, mean = 338 ms; SD = 31 ms) and F0 (mean = 238 Hz). All stimuli were equated at the global root-mean-square (RMS) level.

**Table 1:** Eight distinct phonetic conditions were established.

	**Feature contrast**	**Background**	**Target**
Vowel contrasts	Place	Back: pu, bu, tu, du, po, bo, to, do	Front: py, by, ty, dy, pø, bø, tø, dø
		Front: py, by, ty, dy, pø, bø, tø, dø	Back: pu, bu, tu, du, po, bo, to, do
	Height	Open : po, bo, to, do, pø, bø, tø, dø	Closed: py, by, ty, dy, pu, bu, tu, du
		Closed: py, by, ty, dy, pu, bu, tu, du	Open: po, bo, to, do, pø, bø, tø, dø
Consonant contrasts	Place	Labial: py, pø, pu, po, by, bø, bu, bo	Coronal: ty, tø, tu, to, dy, dø, du, do
		Coronal: ty, tø, tu, to, dy, dø, du, do	Labial: py, pø, pu, po, by, bø, bu, bo
	Voicing	Voiced: by, bø, bu, bo, dy, dø, du, do	Voiceless: py, pø, pu, po, ty, tø, tu, to
		Voiceless: py, pø, pu, po, ty, tø, tu, to	Voiced: by, bø, bu, bo, dy, dø, du, do

In every condition, background syllables were played in a random sequence. During a “change” trial, one randomly selected “target” syllable replaced a background syllable. Conversely, in a “no-change” trial, only a background syllable was played. The specific target syllables were determined based on the phonetic condition being tested.

**Figure 1:**
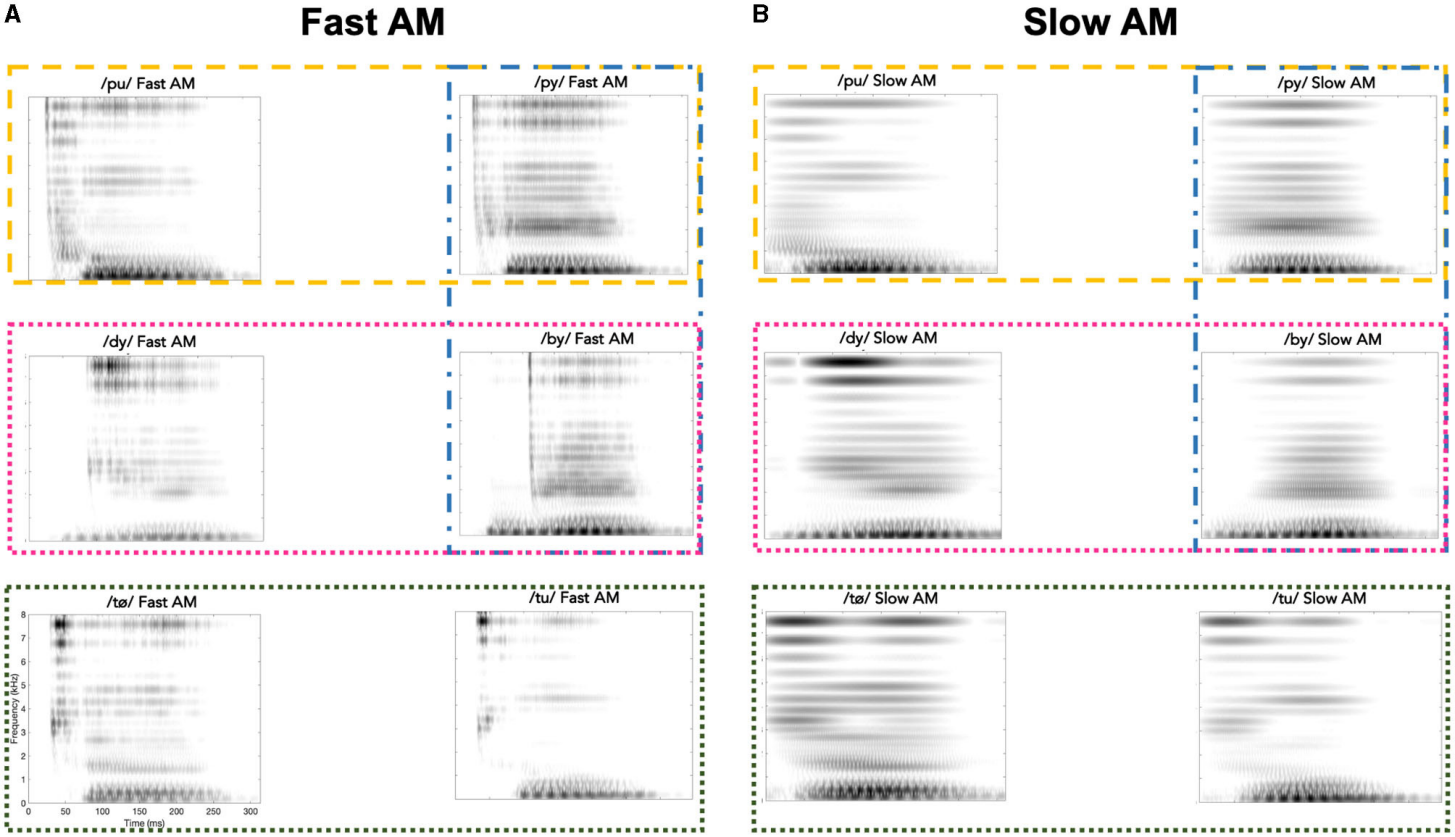
Spectrograms of exemplary syllable tokens in the two vocoded conditions: **(A)** for Fast AM condition and **(B)** for Slow AM condition. The syllables /pu/, /dy/, /tø/ are represented on the left columns, and the syllables /py/, /by/, /tu/ on the right columns. The four different phonetic feature contrasts are illustrated by colored rectangles (vowel place, vowel height, consonant place, consonant voicing by dashed yellow lines, dotted dark green lines, dotted pink lines and dash-dotted blue lines, respectively).

The original stimuli were processed by two vocoders to alter the spectro-temporal modulations. Tone-excited vocoders were used instead of noise-excited vocoders, because they distort speech AM cues less (e.g., Kates, 2011). In each vocoder condition, the original speech signal was passed through a bank of 32 2nd-order gammatone filters (Patterson, 1987; Gnansia et al., 2009), each 1-ERB wide with center frequencies (CFs) uniformly spaced along an ERB scale ranging from 80 to 8,020 Hz. The Hilbert transform was then applied to each bandpass filtered speech signal to extract the AM component and FM carrier. The FM carrier in each frequency band was replaced by a sine wave carrier with a frequency at the CF of the gammatone filter and random starting phase. The AM component was low-pass filtered using a zero-phase Butterworth filter (36 dB/octave roll off) with a cutoff frequency set to either ERBN/2 (Fast AM Condition) or 8 Hz (AM < 8 Hz condition, Slow AM Condition). Each tone carrier was multiplied by the corresponding filtered AM function. The narrow-band speech signals were finally added up and the level of the wideband speech signal was adjusted to have the same RMS value as the input signal. Figure 1 represents the spectrograms of exemplary tokens illustrating the four different contrast types (i.e., vowel place, vowel height, consonant place, consonant height) in the two vocoded conditions.

### 2.3 Procedure, material, and apparatus

Infants were tested using an observer-based psychophysical procedure (Werner, 1995). This procedure is similar to the classical head-turn conditioning procedure used in psycholinguistic studies (Werker et al., 1997), with two key differences: (1) any behavioral change from the infants is considered a response to a sound change, not just head turns, and 2) false alarms are recorded to ensure that the detected behavioral change corresponds to a sound change (Olsho et al., 1987). During testing, infants sat on a caregiver's lap with an assistant inside a sound-attenuating booth. A TV screen was placed on the right of the participant. The infant listened to sounds through an insert earphone (ER-2), calibrated to deliver the sounds at 65 dB SPL, ensuring that none of the adults involved could hear the stimuli presented to the infant. The caregiver was instructed to avoid interacting with the infant.

The experimenter (or “observer”, who was the same for all infants) sat outside the booth and observed the infant through a one-way mirror. A microphone inside the booth enabled the experimenter to listen to the infant and assistant, and a microphone outside the booth allowed the experimenter to communicate with the assistant who was wearing headphones. The assistant listened to the experimenter's instructions and manipulated toys silently to keep infants facing midline. A computer controlled the experiment. Adult participants were tested using the same setup, except that they sat alone in the booth. An advantage of the observer-based procedure over procedures previously used to assess infants discrimination of vocoded speech is that adults can be tested in the same procedure as a basis of comparison.

The participant heard repeated, randomly selected tokens from one “background” category, separated by silences of 800 ms. Each infant participant was tested in only one phonetic condition from Table 1, so that 10 infant participants completed the task in each age group and phonetic condition. Each adult was tested in 2 conditions (one Vowel, one Consonant) in a random order, varying which vowel and consonant condition was presented, so that 10 adult participants completed the task in each phonetic condition.

Test trials were initiated by the experimenter at moments when the participant was quietly listening to the syllables from the background category and facing midline. There were two trial types: on change trials, a syllable from the target category was presented once, while on no-change trials, a syllable from the background category was presented once. On each trial, the experimenter, blind to trial type, had 4 s from trial onset to decide whether the participant had reacted, that is, had produced a behavior during that time window, and to press a button if such a behavioral change was detected. For infants, the behaviors coded as response by the experimenter varied from infant to infant, and commonly observed behaviors included eye movements, increases and decreases in body movement, and facial expressions. Adults were instructed to raise their hand when they detected a change in the sounds. Computer feedback was provided to the experimenter at the end of a trial to indicate hit, miss, correct rejection, or false alarm. Participants responses were automatically reinforced with the presentation of a video for 4 s only if the participant correctly reacted during a change trial.

The experiment consisted of 3 phases, a demonstration phase and 2 test phases. The phases were presented in a fixed sequence: Participants were required to reach criterion on one phase before moving to the next. In the demonstration phase and in the first test phase, the stimuli were from the Fast AM Condition. In the second test phase, the stimuli were from the Slow AM condition.

The purpose of the demonstration phase was to familiarize the participant with the association between the reinforcer (i.e., video) and the target sounds. In this phase, the probability of a change trial was 0.80, and the reinforcer was activated after every change trial regardless of the participant's response. The demonstration phase, which lasted a maximum of 12 trials, ended as soon as the participant had responded correctly to 1 change trial (hence had reacted to the category change) and to 1 no-change trial (hence had not reacted to the lack of category change).

In the following test phases, change and no-change trials were presented in random order, with the probability of change and no-change trials being 0.5. The criterion to end the test phase was evaluated on sliding windows of 10 trials, and corresponded to responding correctly on at least 4 out of 5 change trials and at least 4 out of 5 no-change trials, which corresponds to a hit rate of more than 80% and a false alarm rate of <20%. If the criterion was not reached within a maximum number of trials, the session ended and a new session was started after a short break. If the participants could not reach the criterion within a maximum number of sessions, the participant was judged to be unable to complete the phase. In the Fast AM test phase, the maximum number of trials was 40 and the maximum number of sessions was 4; in the Slow AM test phase, the maximum number of trials was 32 and maximum number of sessions was 3 to minimize the effect of training (Cabrera and Werner, 2017).

**Figure 2:**
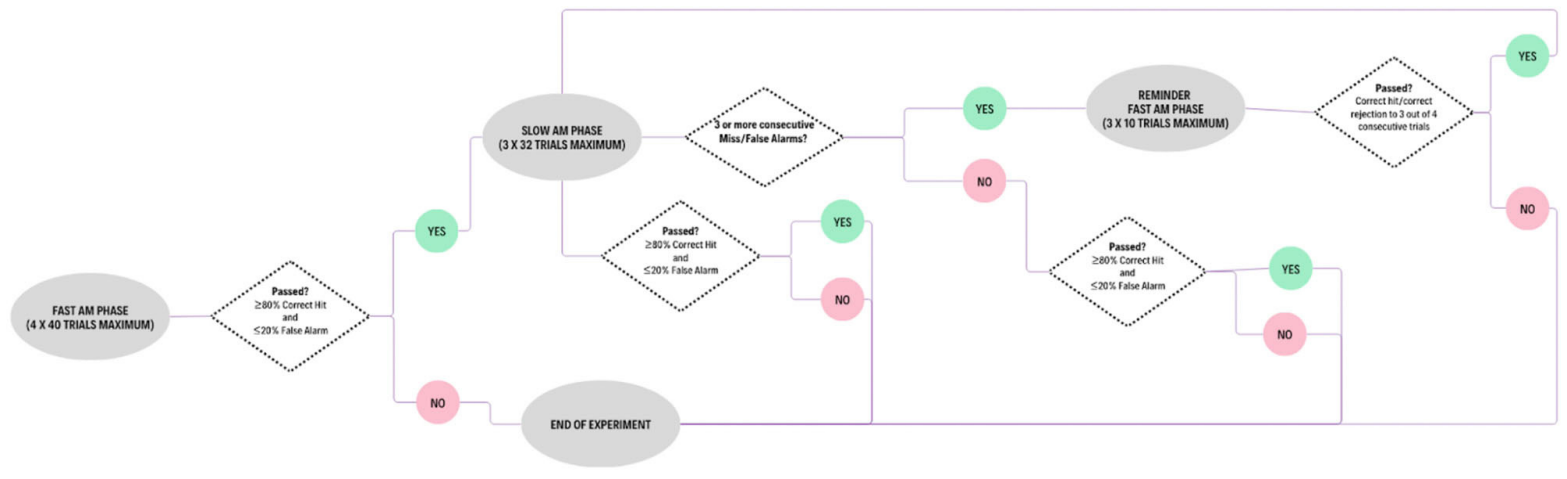
Schematic representation of experimental procedure.

To accommodate the anticipated difficulty in the Slow AM condition, a reminder procedure, similar to the one used by Clarkson and Clifton (1995), was used to assess whether an infant failure was due to factors such as sleepiness or boredom rather than an inability to discriminate. Figure 2 outlines the different scenarios in which the experiment could play out. If a participant responded incorrectly on three consecutive trials in the Slow AM test phase (responding to no-change trials or not responding to change trials), stimuli were presented from the previously completed (and thus succeeded) Fast AM Condition. Up to 10 trials of such “reminder” trials were presented, and if the participant responded correctly on three out of four consecutive trials, the participant returned to the Slow AM phase. If this criterion was not met, the session was discontinued, and infants were given a short break or returned on another day for a new session. Additionally, we ensured that infants were given frequent breaks during the testing process, whenever they appeared to need them, allowing them time to play inside the testing booth, feed, or crawl around as needed. If a participant reached criterion in the Fast AM Condition and reached criterion in three reminder periods without reaching criterion in the Slow AM Condition in three sessions, the participant was judged to be unable to discriminate the phonetic contrast based on the slow AM cues. Because the infant could still perform the discrimination in the Fast AM reminder trials, we could then conclude that the infant's failure in the Slow AM condition did not result from fatigue or loss of interest. As data collection is in line with the infants' individual rhythms, and that infants are more active in such a procedure compared to passive looking time recording procedures, we observed low attrition rates (see Table 2), which are comparable with previous studies using this technique (Olsho et al., 1987; Cabrera and Werner, 2017). It is important to note that, infant testing was completed in one or two visits (lasting around 60 minutes each) on 2 separate days within a 2-week period, which helped to adapt to the infants states. Adult testing was completed in one visit lasting around 60 minutes.

**Table 2:** Breakdown of participants' success rates across different age groups for the different phonetic conditions.

**Age group**	**Phoneme**	**Category**	**Fast AM**	**Slow AM**	**Change in proportion (Slope)**
6-month-olds	Vowels	Overall	16 of 20 (80%)	9 of 16 (56%)	-0.238
		Place	9 of 10 (90%)	4 of 9 (44%)	-0.456
		Height	7 of 10 (70%)	5 of 7 (71%)	0.014
	Consonants	Overall	18 of 20 (90%)	14 of 18 (78%)	-0.122
		Place	8 of 10 (80%)	5 of 8 (63%)	-0.175
		Voicing	10 of 10 (100%)	9 of 10 (90%)	-0.1
10-month-olds	Vowels	Overall	14 of 20 (70%)	10 of 14 (72%)	0.014
		Place	8 of 10 (80%)	6 of 8 (75%)	-0.05
		Height	6 of 10 (60%)	4 of 6 (67%)	0.067
	Consonants	Overall	15 of 20 (75%)	8 of 13 (53%)	-0.217
		Place	5 of 10 (50%)	0 of 3 (0%)	-0.5
		Voicing	10 of 10 (100%)	8 of 10 (80%)	-0.2
Adults	Vowels	Overall	All 20 (100%)	All 20 (100%)	0
		Place	10 of 10 (100%)	10 of 10 (100%)	0
		Height	10 of 10 (100%)	10 of 10 (100%)	0
	Consonants	Overall	All 20 (100%)	18 of 20 (90%)	-0.1
		Place	10 of 10 (100%)	8 of 10 (80%)	-0.2
		Voicing	10 of 10 (100%)	10 of 10 (100%)	0

The main dependent variable analyzed was the proportion of participants who reach success criterion in each phonetic category in each test phase (Fast versus Slow AM). The probability for participants to succeed in the Fast AM condition and then in the Slow AM condition was compared across age groups (6 months versus 10 months versus adults) and (1) phonetic conditions (Vowel versus Consonant) and 2) phonetic features (Vowel Place versus Vowel Height; Consonant Place versus Consonant Voicing). In order to take into account the fact that participants who failed in the Fast AM condition were not tested in the Slow AM condition, we used a modified logistic regression approach called survival analysis to compare the proportion of participants reaching criterion (“survival”) according to age and phonetic condition (or phonetic features). This analysis calculates a survival function for each group representing the cumulative probability that a participant who started the experiment reached criterion in each vocoder condition. The log-rank test for equality, a nonparametric statistic, was used to compare the survival functions for infants and adults, and for vowels and consonants. When a significant difference was found between functions, it meant that either the slope of the function was different between groups (i.e., groups were affected differently by the vocoders if the slopes were not parallel), or the proportion of participants succeeding in either or both vocoder conditions was different between groups (and this was further assessed using χ^2^ tests). Survival function is a non-parametric test well-suited for analyzing smaller sample sizes and effective in discerning temporal patterns, particularly in the non-independent Time 1 and Time 2 conditions of the Fast and Slow tests. Additionally, as a secondary analysis, we used logistic regression to compare the proportion of infants succeeding in the Fast condition across conditions and groups, as well as the proportion succeeding in the Slow condition. It is important to emphasize that these analyses serve as *post-hoc* analyses to provide a better understanding of the differences highlighted by the primary survival function analyses, but given the small number of participants in these exploratory analyses (max *N* = 10), results can only be seen as indications to be further tested in future research.

Additionally, for each age group and phonetic conditions, we compared the number of trials needed to achieve success in each vocoder condition, a metric often used as a measure of processing difficulty in infant studies (Clarkson et al., 1988; Clarkson and Clifton, 1995; Lau and Werner, 2012), using linear models (LM). These analyses thus explored whether infants and adults were able to detect (1) Vowel (Place and Height) and (2) Consonant (Place and Voicing) feature categories when FM is reduced and also when faster AM is reduced.

## 3 Results

### 3.1 Survival function analyses comparing vowels vs. consonants

**Figure 3:**
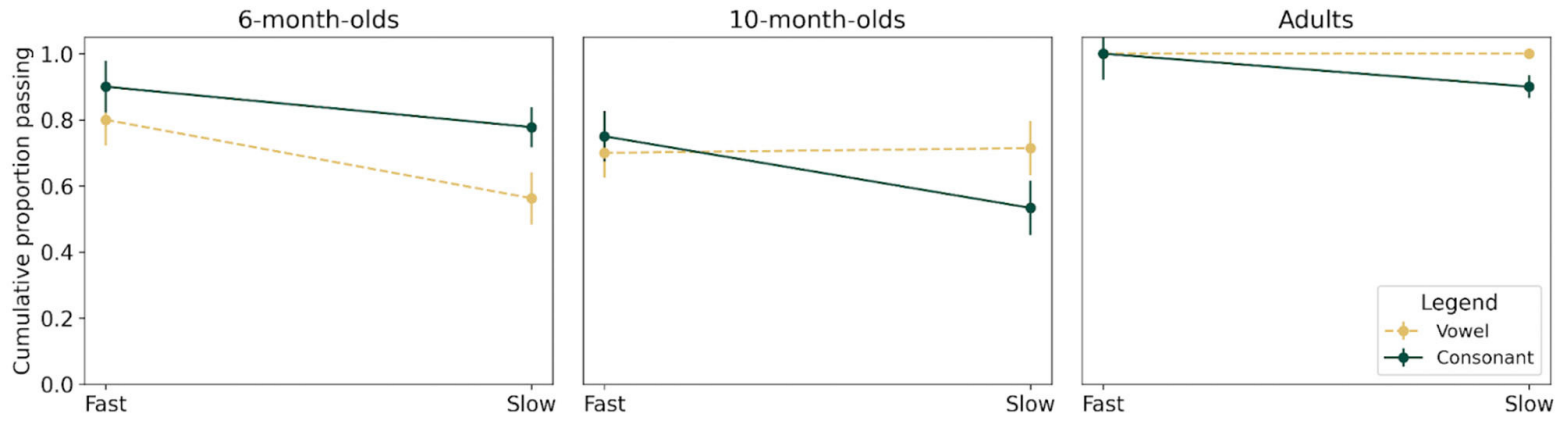
Overall survival plots between Fast and Slow AM conditions (on the x-axis) for Vowel (dashed yellow lines) and Consonant features (solid dark green lines) for 6-month-olds, 10-month-olds, and adults (in each panel). Error bars are standard errors from Kaplan-Meier analysis.

The proportion of participants who reached the 80-20 criterion (*d'* = 1), considered as a measure of detection success, is represented in Figure 3 for each age group and in both vocoder conditions for consonant and vowel categories. See Table 2 for a summary of survival functions for all conditions.

The probability for participants to succeed in the Fast AM condition and then in the Slow AM condition was compared across age groups and phonetic conditions using survival analyses. When comparing all six survival functions defined by Age and Phonetic condition (illustrated in Figure 3), the functions were significantly different [χ^2^(5) = 38.60, *p* < 0.001]. Follow-up analyses were conducted first comparing the functions for Vowels versus Consonants within each Age group. A marginally significant difference was observed at 6 months [χ^2^(1) = 3.10, *p* = 0.08], and no significant difference was observed in the other two age groups [10-month-olds: χ^2^(1) = 0.10, *p* = 0.80; adults: χ^2^(1) = 2.10, *p* = 0.20], suggesting that the detection of vowel or consonant change was affected similarly by vocoding in the three groups. In other words, a similar proportion of participants reached criterion in the Fast and then in the Slow AM condition when exposed to vowel or to consonant change.

The next analyses investigated age effects within each Phonetic Condition (Vowels or Consonants). For Vowels, the functions were not significantly different between 6-month-olds and 10-month-olds [χ^2^(1) = 0.10, *p* = 0.80]. However, there was a significant difference between 6-month-olds and adults [χ^2^(1) = 18.70, *p* < 0.001] because fewer 6-month-olds reached criterion in both conditions [Fast AM: χ^2^(1) = 4.44, *p* = 0.035; Slow AM: χ^2^(1) = 10.86, *p* = 0.001] compared to adults. Moreover, while adults performed at ceiling, 6-month-olds showed a decrease from 80% to 56% between the Fast and Slow conditions when detecting vowel changes. There was also a significant difference in survival functions between 10-month-olds and adults [χ^2^(1) = 19.20, *p* < 0.001], again because fewer 10-month-olds reached criterion in both conditions [Fast AM: χ^2^(1) = 7.06, *p* = 0.008; Slow AM: χ^2^(1) = 6.48, *p* = 0.011] compared to adults, but 10-month-olds showed similar proportions of success in both vocoder conditions (70% at Fast; 72% at Slow).

For Consonants, functions showed a significant difference between 6-month-olds and 10-month-olds [χ^2^(1) = 4.90, *p* = 0.03] and further comparisons between the distribution of succeeding participants showed no significant difference in the Fast AM condition [χ^2^(1) = 1.56, *p* = 0.21] and a trend for fewer 10-month-olds succeeding in the Slow AM condition compared to 6-month-olds [χ^2^(1) = 2.20, *p* = 0.14]. While 6-month-olds showed a decrease from 90% to 78%, 10-month-olds showed a decrease from 75% to 53% suggesting that the detection of consonant change was more affected by vocoding at 10 months than at 6 months (see Figure 3). A significant difference was also found between 6-month-olds and adults [χ^2^(1) = 18.70, *p* < 0.001], related to the fact that overall fewer 6-month-olds reached criterion in both conditions [Fast AM: χ^2^(1) = 4.44, *p* = 0.035; Slow AM: χ^2^(1)=10.86, *p* = 0.001]. Moreover, a significant difference is observed between 10-month-olds and adults [χ^2^(1) = 16.00, *p* = < 0.001], and fewer 10-month-olds reached criterion in both conditions [Fast AM: χ^2^(1) = 5.71, *p*= 0.017; Slow AM: χ^2^(1) = 6.03, *p* = 0.014] compared to adults.

In summary, no difference was observed between 6- and 10-month-olds for vowel change detection, but 10-month-olds were more affected by vocoding than 6-month-olds for consonant change detection. For both consonant and vowel change detection, fewer 6- and 10-month-olds succeeded compared to adults in both vocoder conditions.

### 3.2 Exploratory survival function analyses comparing subcategory of vowels and consonants

**Figure 4:**
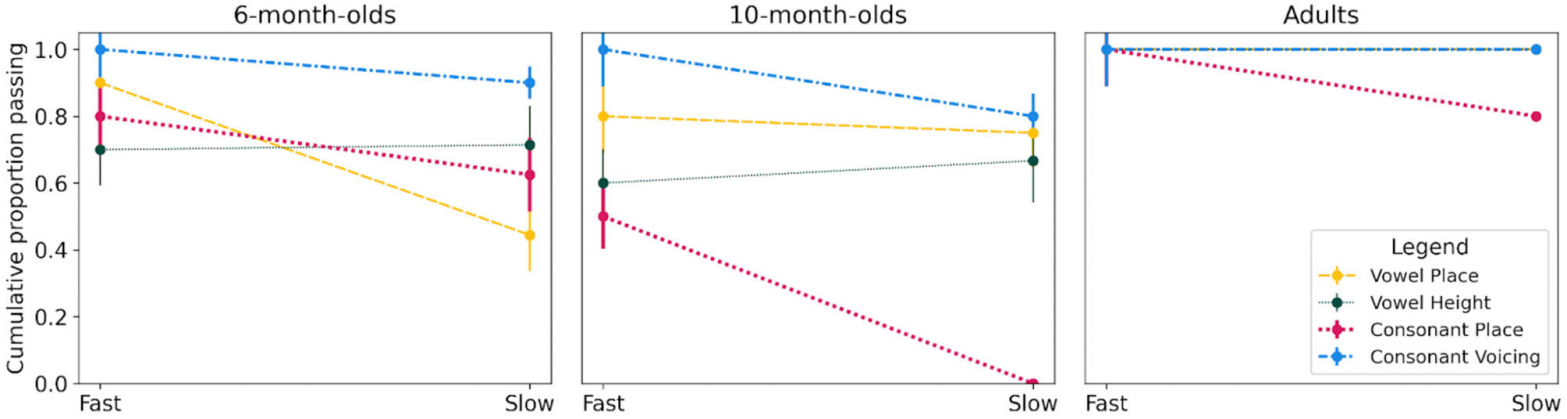
Survival plots between Fast and Slow AM conditions (on the x-axis) for Vowel (Place and Height, dashed yellow lines vs dotted dark green lines, respectively) and Consonant features (Place and Voicing, dotted pink lines vs dash-dotted blue lines, respectively) for 6-month-olds, 10-month-olds, and adults (in each panel). Error bars are standard errors from Kaplan-Meier analysis.

Next, as an exploratory analysis, given the limited sample size of only 10 participants per subgroup, we compared survival functions for vowel features (place versus height) and consonant features (place versus voicing) to assess whether the different phonetic categories rely differently upon temporal cues as a function of age. These functions are represented in Figure 4.

#### 3.2.1 Vowels

When comparing all six survival functions defined by Age and Vowel Feature (3 ages x 2 features), a significant difference was found [χ^2^(5) = 23.00, *p* < 0.001]. To understand this difference, we first explored the impact of Features for each age group separately. No significant differences were found for the 6-month-olds [χ^2^(1) = 0.10, *p* = 0.70], the 10-month-olds [χ^2^(1) = 1.50, *p* = 0.20], or the adults [χ^2^(1) = 0.00, *p* = 1.00], suggesting that detection of vowel height and vowel place change in all age groups was affected similarly by vocoding (i.e., in each age group, a similar proportion of participants reached criterion in the Fast and then in the Slow AM condition when exposed to either vowel height or place change).

The next comparisons addressed differences between Age groups for each vowel feature. For vowel place, a main effect of Age was found [χ^2^(2) = 8.80, *p* = 0.01], and pairwise comparisons revealed no significant effect between the two infant groups [χ^2^(1) = 0.10, *p* = 0.70], but a significant difference between 6-month-olds and adults [χ^2^(1) = 9.00, *p* = 0.003], and between 10-month-olds and adults [χ^2^(1) = 6.80, *p* = 0.009]. The differences were related to significant lower proportions of 6-month-olds reaching criterion in the Slow AM condition compared to adults [Fast AM: χ^2^(1) = 1.05, *p* = 0.305; Slow AM: χ^2^(1) = 7.54, *p* = 0.006], and to marginally lower proportions of 10-month-olds reaching criterion in the Slow AM condition compared to adults [Fast AM: χ^2^(1) = 2.22, *p* = 0.136; Slow AM: χ^2^(1) = 2.81, *p* = 0.093]. Specifically, for 6-month-olds success rates decreased from 90% to 44% and for 10-month-olds performance decreased from 80% to 75% between Fast and Slow conditions.

For vowel height, age influenced the functions [χ^2^(2) = 12.40, *p* = 0.002], and subsequent pairwise comparisons revealed no significant difference between 6- and 10-month-olds [χ^2^(1) = 0.40, *p* = 0.50], but a significant difference between 6-month-olds and adults [χ^2^(1) = 9.40, *p* = 0.002] and between 10-month-olds and adults [χ^2^(1) = 12.30, *p* < 0.001]. These differences were related to marginally lower proportions of 6-month-olds reaching criterion in both conditions [Fast AM: χ^2^(1) = 3.53, *p* = 0.06; Slow AM: χ^2^(1) = 3.24, *p* = 0.070] compared to adults. Similar but significant effects were observed between 10-month-olds and adults [Fast AM: χ^2^(1) = 5.00, *p* = 0.03; Slow AM: χ^2^(1) = 3.81, *p* = 0.05]. Here, again, adults perform at ceiling while both infant groups show an overall lower success rate, albeit similarly affected in the Fast (6 months: 70%; 10 months: 60%) and Slow (6 months: 71%; 10 months: 67%) conditions.

In summary, no difference was observed between vowel height and vowel place in any group. However, fewer 6- and 10-month-olds succeeded in detecting vowel place and vowel height change under the current vocoder conditions compared to adults. For vowel place, a lower proportion of infants succeeded the detection compared to adults in the Slow AM condition, while for vowel height, lower proportions were observed in both vocoder conditions.

#### 3.2.2 Consonants

When comparing the six survival functions defined by Age and Consonant Feature (3 ages × 2 features), a significant difference was found [χ^2^(5) = 48.00, *p* < 0.001]. To understand this difference, subsequent comparisons assessed the impact of Features for each age group separately. There were significant differences between the survival functions for place and voicing for 6-month-olds [χ^2^(1) = 5.70, *p* = 0.02] and for 10-month-olds [χ^2^(1) = 17.70, *p* < 0.001]. A marginal difference emerged for adults [χ^2^(1) = 2.10, *p* = 0.10] because two participants only failed to detect place change in the Slow AM condition. At 6 months, the comparison of participants reaching the criterion between voicing and place contrasts did not show statistical significance in either the Fast [χ^2^(1) = 2.22, *p* = 0.136] or Slow AM conditions [χ^2^(1) = 1.94, *p* = 0.163]. This suggests that the observed differences in survival functions for voicing and place contrasts are not statistically significant. However, there is an overall difference in the proportion of participants meeting the criterion, with a higher proportion for voicing (90%) compared to place (63%) in both conditions. At 10 months, this difference is characterized by a lower proportion of participants able to detect the place change compared to the voicing change in both vocoder conditions [Fast: χ^2^(1) = 6.67, *p* = 0.01; Slow: χ^2^(1) = 8.57, *p* = 0.003] and by a steeper decrease in proportion of participants reaching criterion from Fast to Slow AM conditions for place than for voicing. For place, 10-month-olds showed a decrease between the two vocoder conditions from 50% to 0% whereas for voicing the decrease was much smaller from 100% to 80%.

Next, we addressed differences between Age groups for each consonant feature. For place, a significant Age effect was observed on the survival functions, [χ^2^(2) = 18.50, *p* < 0.001], and 2-by-2 comparisons revealed significant differences between 6-month-olds and adults [χ^2^(2) = 3.70, *p* = 0.05] and 10-month-olds and adults [χ^2^(2) = 17.70, *p* < 0.001], with no significant difference between the two infant groups [χ^2^(1) = 0.40, *p* = 0.50]. These differences were related to lower proportions of 10-month-olds reaching criterion in both vocoder conditions [Fast AM: χ^2^(1) = 6.67, *p* = 0.01; Slow AM: χ^2^(1) = 8.57, *p* = 0.003] compared to adults. Moreover, 10-month-olds showed a stark decrease from 50% to 0% success rates between the two conditions, while adults went from a 100% success rate to an 80% one. Similar but non-significant trends were found in the Fast AM condition between 6-month-olds and adults [Fast AM: χ^2^(1) = 2.22, *p* = 0.14; Slow AM: χ^2^(1) = 0.68, *p* = 0.41], suggesting an overall effect of less 6-month-olds reaching criterion (71.5% compared to adults (90%). For voicing, no significant effect of Age was found [χ^2^(2) = 2.10, *p* = 0.30].

In summary, a difference in the proportion of participants able to succeed detection between the Fast AM and the Slow AM conditions was observed between voicing and place for both infant groups only. Moreover, while no difference was observed between either infant groups or adults for voicing detection, fewer 6- and 10-month-old infants were able to reach success in both vocoder conditions for place compared to adults, and 10-month-olds showed a strong decrease between the two vocoder conditions.

### 3.3 Linear Models comparing the numbers of trials to reach criterion

**Figure 5:**
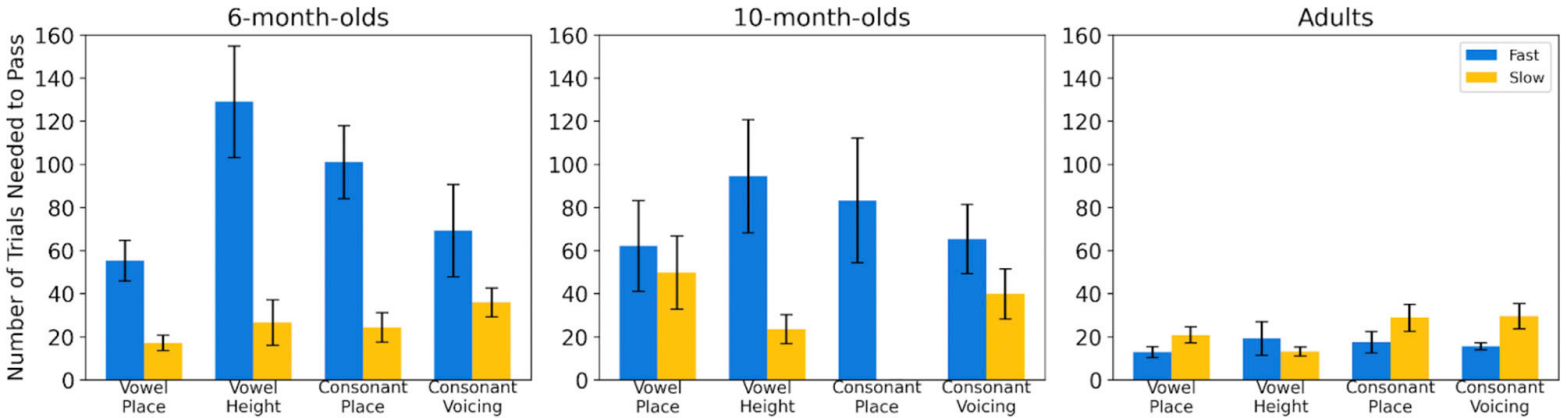
Average number of trials (and standard errors) needed to succeed Fast (blue bars) and Slow (yellow bars) phases in each phonetic feature condition (x-axis) for 6-month-olds, 10-month-olds and adults (in each panel).

In order to further understand whether task difficulty was affected by Vocoder condition (Fast AM versus Slow AM), Phonetic Condition (Vowels vs Consonants) or Phonetic Feature (vowel place versus vowel height versus consonant place versus consonant voicing) and Age (6 months vs 10 months), Linear models were used to analyze the average number of trials needed to succeed the task (see Figure 5). Adults' data were analyzed in individual models to assess the effect of Phonetic Conditions or Phonetic Features. All analyses were conducted in R (version 4.3.1, R Core Team, 2019). We fitted linear models using the lm function.

In the Fast AM test phase, the maximum number of trials was set at 40, with a limit of 4 sessions. Conversely, in the Slow AM test phase, we limited the number to 32 trials and a maximum of 3 sessions. In the following analysis, the average number of trials required to achieve the success criterion thus corresponds to the average of the total number of trials over the sessions required by each infant in each phase. This analysis was conducted first for the Fast AM condition, followed by the Slow AM condition.

For infants, we used the following Linear model to analyze the average number of trials needed to achieve the success criterion, first in the Fast AM, then in the Slow AM condition:


$$\begin{array}{c} \text{Average Number of Trials}\\ \sim \text{Age}*\text{Phonetic Condition }\left(\text{Vowel}/\text{Consonant}\right) \end{array}$$

For the Fast AM condition, the ANOVA failed to find significant effects of Age [F_(1,59)_ = 0.61, *p* = 0.436], Phonetic Condition [F_(1,59)_ = 0.08, *p* = 0.774], or the Age x Phonetic Condition interaction [F_(1,59)_ = 0.001, *p* = 0.975]. Likewise, for the Slow AM condition, the ANOVA failed to find significant effects of Age [F_(1,37)_ = 1.91, *p* = 0.176], Phonetic Condition [F_(1,37)_ = 0.42, *p* = 0.521] or the Age x Phonetic Condition interaction [F_(1,37)_ = 0.27, *p* = 0.605].

For Adults, we used the following Linear Models to evaluate the average number of trials needed to pass the Fast and Slow conditions:


$$\begin{array}{c} \text{Average Number of Trials}\\ \sim \text{Phonetic Condition }\left(\text{Vowel}/\text{Consonant}\right) \end{array}$$

For the Fast condition, the ANOVA failed to find a significant effect of Phonetic Condition [F_(1,38)_ = 0.01, *p* = 0.926]. For the Slow condition, the ANOVA revealed a significant effect of Phonetic Condition [F_(1,36)_ = 7.09, *p* = 0.012], and *post-hoc* analyses revealed that it took more trials to achieve success for consonants (29 trials in average) than for vowels (17 trials in average), which indicates a greater level of difficulty for consonants than vowels. Given the significant effect of Phonetic Condition, follow up analyses were conducted comparing average number of trials within the vowel and consonant categories, using the following LM:


$$\begin{array}{c} \text{Average Number of Trials \textasciitilde{} Phonetic Feature} \end{array}$$

For vowels, the ANOVA found a marginally significant effect of Phonetic Feature [F_(1,18)_ = 3.30, *p* = 0.086].

## 4 Discussion

The present study explores the reliance of 6-month-olds, 10-month-olds and adults upon spectro-temporal modulations of speech when categorizing consonant and vowel contrasts based on different phonetic features (for vowels: place and height; for consonants: place and voicing). Results show that 6-month-olds, 10-month-olds and adults are able to use AM cues, and even the slowest AM cues only (<8 Hz) for both vowel and consonant categorization. Indeed, in the Fast AM condition, in which FM cues were replaced but original AM cues were preserved in a large number (N = 32) of spectral bands, the overall proportion of participants succeeding the detection of vowels and consonants averaged together, was 85% in 6-month-olds, 73% in 10-month-olds, and 100% in adults. This first result establishes that at the three ages, the participants could successfully detect the vowel/consonant changes based solely on AM cues. It suggests that, in quiet, FM is not necessary for phonetic categorization for the majority of 6-month-olds, 10-month-olds, or adults. Moreover, among participants who succeeded in the Fast AM condition, the overall success rates in the Slow AM condition, in which only the slowest AM cues (<8 Hz) were preserved, were 67% in 6-month-olds, 63% in 10-month-olds, and 95% in adults. This again establishes that at the three ages, most of the participants who could successfully detect the vowel/consonant changes using only AM cues could also detect those changes based on Slow AM cues only.

Our adult results show that although adults required more trials to reach success criterion when detecting consonant compared to vowel changes in the Slow AM condition, they were at ceiling in both vocoder conditions for all phonetic feature contrasts tested. This indicates that FM cues are not necessary for phoneme processing in adults, and that they are able to rely solely on slow AM cues (<8 Hz). This pattern is similar to what was found in previous studies with adult listeners showing near perfect identification or discrimination scores on the basis of slow AM cues in quiet (Drullman et al., 1994a,b; Cabrera and Werner, 2017). Moreover, the higher number of trials required when detecting consonants based on the slowest AM cues is also consistent with previous studies showing a stronger impact of temporal reduction when processing consonants compared to vowels (Xu et al., 2005).

Our infant results suggest that slow AM cues (<8 Hz) provide enough information for most infants to successfully process the phonetic contrasts used in our task. They are consistent with previous infant experiments showing that 3- and 6-month-olds are able to discriminate consonant place and voicing on the basis of the original AM or slow AM cues (Bertoncini et al., 2011; Cabrera et al., 2013, 2015a; Cabrera and Werner, 2017). Crucially though, they add new data for vowel processing, as only one previous vocoder study had been conducted testing discrimination of a very large vowel contrast (/a/ versus /i/, Warner-Czyz et al., 2014). Here we demonstrate, for the first time, that both 6- and 10-month-olds can process vowel place and vowel height in conditions of reduced FM and AM cues. This aligns with evidence that young infants possess auditory mechanisms with relatively mature auditory temporal and spectral resolution (Folsom and Wynne, 1987; Spetner and Olsho, 1990; Levi and Werner, 1996).

### 4.1 Contributions of temporal cues to the categorization of vowels vs. consonants

Importantly, no overall difference was observed between the consonant and vowel conditions (that is, when the two phonetic feature conditions are averaged) in any age group. In other words, the proportion of participants succeeding the task was not different when they had to detect a change in vowel or a change in consonant. This finding suggests that the ability in detecting these changes are affected similarly by temporal degradation. However, differences between age groups appeared, indicating that with age the reliance upon temporal modulations may differ when processing native vowels and consonants. For vowels, while the 6- and 10-month-old groups did not differ from one another for overall detection in the vocoded conditions, they both independently differed from adults. Contrary to our expectations, we did not observe any significant difference in the reliance upon FM and faster AM cues between 6 and 10 months of age for vowel categorization. However, the number of participants succeeding in detecting the vowel contrasts was significantly lower for both groups of infants compared to adults in both vocoder conditions. More precisely, both 6- and 10-month-olds are more affected by FM degradation compared to adults and are also more affected by faster AM degradation (if they succeeded the Fast AM condition) compared to adults. Six-month-olds also displayed a specific result, that is, they showed a more important decrease of success rate in the Slow AM condition compared to adults. Altogether, these findings reveal that both FM and fast AM cues play a critical role in vowel categorization for both infant age groups, likely due to the role of faster temporal cues in conveying fine spectro-temporal details that are probably important for vocalic processing (Rosen, 1992). The fact that significantly fewer 6-month-olds succeeded the task compared to adults when the faster AM cues are reduced may also suggest that these temporal modulations are important for them to successfully detect vocalic changes.

For consonants, an overall difference was observed between the two infant groups, with 10-month-olds showing a stronger impact of vocoding on consonant detection compared to 6-month-olds, and a significantly greater decline in detection success rates between the Fast AM and Slow AM conditions compared to 6-months (75% and 53% vs. 90% and 78%, respectively). Thus, the degradation of faster AM cues (>8 Hz) appears to strongly affect consonant processing at 10 months of age. This difference in overall consonant processing is contrary to our hypothesis as we expected any difference between the effect of temporal degradation on consonants to be less pronounced in 10-month-olds than in 6-month-olds, the former being more advanced in their speech perceptual attunement to their native consonants. However, as will be further discussed later, this result can be further nuanced based on consonant feature category. It is then possible that while 10-month-olds become more attuned to the consonants of their native language, they are also more impeded by their linguistic experience to rely on the residual AM information. This is in line with previous cross-linguistic studies using vocoders with infants and adults showing that native listeners are more impaired by reduction of fine spectro-temporal cues compared to non-native listeners for consonant and tone processing (Cabrera et al., 2014, 2015a). Moreover, both infant groups independently were more affected by vocoding compared to the adult group, and this, in both vocoder conditions. Importantly, adults showed more robust detection of consonants in degraded conditions compared to 10-month-olds, who are supposed to have started attuning to the consonants of their native language. This different perceptual weight on temporal modulations for consonant change detection suggests that further changes in acoustic processing take place after the onset of perceptual attunement around 10 months. This is congruent with some studies showing that phonological categorization continues to develop until 12 years of age, and that children do not rely on the same acoustic (i.e., spectral or VOT) cues as adults to distinguish between native phonemes (e.g., Lehman and Sharf, 1989; Hazan and Barrett, 2000; Mayo et al., 2003; Nittrouer, 2004; Nittrouer and Lowenstein, 2007).

### 4.2 Contributions of temporal cues to the categorization of phonetic features

In the present experimental design, we also manipulated the phonetic feature to be detected within the vowel and the consonant condition: vowel place versus vowel height, and consonant place versus consonant voicing. In our task, participants heard a string of syllables that varied in consonants and vowels but shared one phonetic feature (for example, back vowels: pu, bu, tu, du, po, bo, to, do), and they were required to react to a new syllable corresponding to a feature change (for example, front vowels: py, by, ty, dy, pø, bø, tø, dø). To perform this task, participants could have discriminated the eight new syllables from the eight syllables presented as background. Alternatively, they could have categorized the syllables according to phonetic features, and discriminate categories of syllables based on the contrasting feature (in the example above, vowel place). If so, albeit exploratory, our findings would add to a small number of studies showing that phonetic features appear to be used in infant processing. In both vocoder conditions, all age groups were able to successfully detect a change in phonetic features on the basis of AM cues. Around 9 to 10 months, infants can form generalizations across different speech segments on the basis of place of articulation (Seidl and Buckley, 2005), they can learn constraints between non-adjacent consonants, but only when the consonants share a phonetic feature (Saffran and Thiessen, 2003) and their phonotactic knowledge appears constrained by phonetic features (Gonzalez-Gomez and Nazzi, 2015). Moreover, between 4 and 7 months, infants' acquisition and generalization of phonological constraints on consonant categories becomes constrained by the fact that those categories are defined by a single phonetic feature (Cristià and Seidl, 2008; Cristià et al., 2011). Our exploratory findings would add another piece of evidence in support of a role of phonetic features in early language processing and acquisition, providing the first piece of evidence of an early use also of vowel features, and that the cues needed to process these features are contained in the AM information.

These findings also reveal that the ability to detect phonetic feature changes was affected differently by vocoder and age of the listeners. The detection of vowel place and vowel height was affected similarly by vocoding in all age groups, meaning that one vocalic feature was not easier to detect than the other when FM or faster AM cues are degraded. However, age differences occurred within each phonetic feature category between infant groups and adults, while no difference was observed between 6- and 10-month-olds. For vowel place detection, 6-month-olds were more affected by the reduction of fast AM cues compared to adults, while 10-month-olds were overall worse than adults in both vocoder conditions (with no further decrease in the proportion of participants succeeding the task in the Slow AM condition). For vowel height, both 6-month-olds and 10-month-olds showed lower success rates compared to adults in both vocoder conditions. These findings may suggest that at 6 months infants require faster AM cues (> 8 Hz) to efficiently detect changes in vowel features. Importantly, the vocoders used in the current experiment did not drastically affect the original formants of the vowels, as the spectral resolution of the vocoded signals was pretty high including 32 spectral bands. Furthermore, in adults, it has been shown that vowel perception is more affected by spectral degradation than by temporal degradation (Xu and Pfingst, 2003). In our results, infants are more sensitive to a degradation of the temporal modulations of vowels compared to adults, suggesting a stronger perceptual weight on relatively fast temporal modulations in infancy even for vowel processing. FM cues and faster AM cues convey information about the spectrum of speech and thus, about the formant pattern (Rosen, 1992). It is possible that infants from 6 to 10 months of age rely more strongly on these temporal cues compared to adults, and are more sensitive to subtle modification of the speech spectrum, like older children have been shown to be more sensitive to the dynamic spectral structure in vowel identification (Nittrouer and Lowenstein, 2007). For consonant features, a different pattern was observed, that is, both infant groups were less affected by temporal cue reduction for voicing contrasts compared to place of articulation contrasts. No age difference was observed in the proportion of infants and adults succeeding the detection of voicing change when FM cues were degraded or when faster AM cues were degraded. One result of note is that in the Fast AM condition, all age groups achieved a 100% success rate for voicing contrasts. On the other hand, the detection of place change for consonants was significantly different with age and as a function of the vocoder condition. Significantly less infants, at both 6 and 10 months of age, compared to adults were able to complete the task in the vocoded conditions. Six-month-olds were overall more affected than adults when FM cues and faster AM cues were reduced. Ten-month-olds were strongly affected by reduction of fast AM cues compared to adults as none of the infants tested were able to detect the place change in the Slow AM condition. These findings align with prior adult research which indicated that the identification of place is notably vulnerable to spectro-temporal degradation. For instance, Shannon et al. (1995) highlighted that, unlike other phonetic features, the identification of place suffered in scenarios with limited spectral bands where FM cues were degraded. Drullman et al. (1994a,b) also observed that place of articulation for stop consonants was difficult to identify by adult listeners when reducing FM and faster AM cues. Again, as FM cues convey information about the spectrum, it has been suggested that place is particularly affected by such degradation compared to voicing (Rosen, 1992). In the present task, adults were not impacted by this FM degradation even for place, which reveals that even though the task was implicit (i.e., without direct instruction about what the participants should be attending in the signal), adult listeners were extremely good at detecting any phonetic change even the ones usually more difficult to identify.

In sum, the developmental differences between infants and adults in success rates between the Fast and the Slow AM conditions reveal different reliance upon temporal cues for phonetic perception between infancy and adulthood. To some extent, the present results are consistent with previous behavioral studies showing that younger infants more strongly rely on fast AM (> 0.8Hz) compared to adults. However, Cabrera and Werner (2017) using similar methods and vocoder conditions with 3-month-old infants and young adults showed that less than half of the infants differentiated between consonants (contrasting on either voicing or place) when only slow AM cues were preserved (i.e., Slow AM condition). In the current study, the filtering of faster AM cues did not impact as drastically the success rate of 6-month-olds for consonant detection. This discrepancy might be attributed to the age difference between the two studies, but also to the fact that in the previous study, the same vocalic context /a/ was used when presenting the vocoded syllables, and more consonant types were presented within the background (e.g., /b/, /p/, /d/, /t/, but also /k/, /g/), while in the current study, multiple vocalic contexts are presented /o/, /ø/, /u, /y/, and only two different consonants were presented in the background. Thus, it is possible that infants in the present design might have been able to leverage different mechanisms to compensate for the impact of acoustic temporal degradation on consonant discrimination, potentially due to vowel and consonant variability. Finer differences are then observed in the present study compared to the previous ones that did not find any difference in the detection of voicing and place in such vocoded conditions (Cabrera et al., 2015a; Cabrera and Werner, 2017). The present design may have “helped” infants to detect changes in voicing, not requiring FM or faster AM cues, while it may have impeded their detection of the place contrasts known to be more sensitive to any acoustic degradation (Miller and Nicely, 1955). Moreover, it is important to note that the present stimuli were from the French language where /p/ and /t/ are voiceless and unaspirated, and /b/ and /d/ are pre-voiced, whereas in English /p/ and /t/ are aspirated and /b/ and /d/ are partially voiced. These differences may also contribute to the discrepancy between the two studies. Finally, no significant differences were observed in the proportion of participants succeeding the detection of vowel changes between 6- and 10-month-old infants, but a significant difference emerged for consonant changes. This relates to the specific difficulty of 10-month-olds to detect the place change in the consonant condition when only the slowest AM cues are available. These findings thus suggest a similar weighting of FM and fast AM cues between 6 and 10 months of age for vowel categorization, perhaps because at these two ages infants have already started to attune to their native vowels, but a stronger reliance upon faster AM cues at 10 months for processing some native consonant features. This difference may relate to later onset of perceptual attunement for consonants than vowels. Future studies are required to determine whether differences in the perceptual weight of acoustic temporal cues for consonants are related to some other language milestones for instance lexical acquisition.

### 4.3 Conclusions

The present results indicate that infants, in comparison to adults, are more sensitive to the deterioration of FM and faster AM cues (> 8 Hz). They further indicate that infants between 6 and 10 months of age assign a similar perceptual weight to FM and fast AM cues when categorizing vowels, possibly because they already process vowels in a language-specific way (since they have started to attune to their native language vowels). However, at 10 months, there appears to be a stronger reliance for faster AM cues for consonants, especially when processing place of articulation. This difference between vowels and consonants might be linked to the later onset of infants' perceptual reorganization to consonant sounds, which begins between 6 and 10 months. Altogether, this study underscores the significant role of speech temporal cues in vowel and consonant categorization during infancy and suggests that the ability to rely solely on slow AM cues for phonetic categorization develops later in life.

## Data availability statement

The raw data supporting the conclusions of this article will be made available by the authors, without undue reservation.

## Ethics statement

The studies involving humans were approved by Le comité d'éthique de la recherche (CER)-Paris Descartes. The studies were conducted in accordance with the local legislation and institutional requirements. Written informed consent for participation in this study was provided by the participants' legal guardians/next of kin.

## Author contributions

MH: Conceptualization, Formal analysis, Writing—original draft, Writing—review & editing. TN: Conceptualization, Writing—original draft, Writing—review & editing. LC: Conceptualization, Formal analysis, Writing—original draft, Writing—review & editing.
